# Exploring the Lives of Women Rag Pickers in an Indian Metropolitan City: A Mixed-Methods Cross-Sectional Study on Social and Occupational Determinants Shaping Their Existence

**DOI:** 10.7759/cureus.47464

**Published:** 2023-10-22

**Authors:** Swati Iyer, Harsh Shah, Jay Patel, Vishal Panchal, Shalu Chaudhary, Trushar Parmar

**Affiliations:** 1 Health Policy, Independent Consultant, Mumbai, IND; 2 Public Health Sciences, Indian Institute of Public Health Gandhinagar, Gandhinagar, IND; 3 Public Health Sciences, ​Indian Institute of Public Health Gandhinagar, Gandhinagar, IND; 4 Public Health, Independent Consultant, New Delhi, IND

**Keywords:** india, health policy, occupational health, marginalized population, rag-pickers

## Abstract

Background: Globally, occupational hazards are a concern, especially in waste management. With 31.2% of its population in urban areas, India is confronted with escalating waste management challenges. People worldwide generate about two-thirds of a kilogram of waste daily. Effective solid waste management is crucial due to population growth, changing waste patterns, and rapid urbanisation. It profoundly impacts environmental, resident, and worker health. Rag picking is an informal profession undertaken by a marginalised population of the society, which involves collecting waste from trash cans, streets, and household waste. To assess the burden and the pattern of morbidity, and the occupational factors associated with it, as well as their health-seeking behaviour, the present study was carried out among women rag pickers in Mumbai, India.

Methodology: A cross-sectional descriptive study was conducted through a mixed-method approach in Mumbai's Chembur and Govandi areas, focusing on women rag pickers aged 15 to 49 years. The research was conducted over a period of three months, during which a total of 150 female rag pickers from individual families were identified and included in the study through purposive sampling. The structured questionnaires gathered quantitative data on socio-demographics, health-seeking behaviour, morbidity, and monthly expenses. The qualitative data were collected through focus group discussions with rag pickers, analysing themes related to rag picking as occupational preference and substance usage factors. Ethical approval from the institute and informed consent from each participant were obtained prior to data collection.

Results: Among the cohort of 150 women rag pickers, 67.3% were aged between 15 and 30 years, with 82% belonging to the lower socio-economic class. A notable 43.4% of these women engaged in significant tobacco use, primarily through oral consumption, while about 56.7% of their family members exhibited high substance use, including pan, tobacco, and alcohol. In terms of health-seeking behaviour, 51% refrained from seeking treatment for minor ailments, 29% resorted to home remedies or self-medication, and 20% sought care at hospitals. A morbidity analysis over the past three months revealed prevalent health issues, informing potential interventions. Examination of monthly expenditure patterns unveiled an average income of 9000 INR (130 USD), with a significant 61% allocation towards food and grocery expenses. Qualitative insights indicated that the preference for rag picking was driven by limited alternatives and substance use was influenced by peers and served as a means to cope with stress. These findings underscore distinct health-seeking behaviours, and the unique needs of women rag pickers, providing valuable guidance for targeted policies to enhance their well-being.

Conclusion: These findings underscore the need for targeted interventions to improve the well-being and socio-economic conditions of women rag pickers in India. Universal healthcare coverage, community-based initiatives, and social inclusion are vital for addressing their unique challenges and enhancing their quality of life.

## Introduction

India is confronted with significant challenges in waste management with its growing urban population, as about 31.2% of the population is now living in urban areas. India produces 12 million tons of inert waste from street sweeping and construction and demolition (C&D) annually, which makes up about one-third of municipal solid waste (MSW) in landfills [1]. Municipal bodies in India, according to estimates, can only collect 70-80% of the country's daily average of 62 million tons of waste [2]. On a worldwide scale, each person generates roughly two-thirds of a kilogram of waste per day. The solid waste management system is crucial because of high population growth, rapidly changing waste characterisation and generation patterns, and expanding urbanisation and industrialisation in developing countries [2,3].

Management processes of solid waste not only have an impact on environmental health and the inhabitants’ health but the health of those directly involved in the processes. Rag picking, also known as waste picking or scavenging, is the practice of collecting and sorting recyclable materials from trash cans, streets, and household waste. Rag pickers engage in the random sorting and collection of refuse, then vending their findings to scrap merchants in exchange for monetary compensation, which depends upon the weight and categorisation of the waste materials [4]. Despite the significance of rag picking in terms of resource recovery and waste management, it is predominantly undertaken by underprivileged people, namely, women, due to the limited availability of alternative employment options [5].

Rag pickers are members of a very vulnerable group who are exploited by both fate and society [6]. Waste pickers are engaged in informal employment within the unregulated sector, which poses challenges in accurately quantifying the amount of waste they collect. When using the disposal site, they risk coming across a lot of plastic and iron items, as well as needles, syringes, used condoms, saline bottles, unclean gloves, and other hospital garbage. Therefore, the rag pickers are prone to various diseases and health hazards, owing to their occupation, such as tuberculosis, bronchitis, asthma, pneumonia, dysentery, parasites, and malnutrition [7].

To assess the burden and the pattern of morbidity, and the occupational factors associated with it, as well as their health-seeking behaviour, the present study was carried out among women rag pickers in Mumbai, India.

## Materials and methods

Study design and settings

A cross-sectional descriptive study with a mixed-method approach was conducted in the Chembur and Govandi areas of Mumbai, known for their significant presence of rag pickers. These locations were chosen due to the concentration of rag pickers in these regions.

Study population and sampling

The study population consisted of women rag pickers, residing and rag picking in Chembur and Govandi areas of Mumbai. The participants of the study were selected using purposive sampling, specifically targeting women who work as rag pickers. This was achieved by conducting visits to different public locations that are known to generate solid waste, such as bus stations, railway stations, streets, hospitals, and dumping sites located in densely populated areas of the city. During the sampling process, support from the local social volunteers was received to get in contact with the study samples. The research was conducted over a period of three months, during which a total of 210 female rag pickers from individual families were identified. The majority of these women belonged to the reproductive age category, specifically between 15 and 49 years old, involved in this informal occupation. However, 150 study samples provided the consent to be included in the study.

Data collection method

The data collection was undertaken by mixed methods involving the quantitative and qualitative interviews of the study participants at a suitable location, ensuring a comfortable and conducive environment for open dialogue. First, the quantitative data were collected through structured, pre-tested questionnaires. The questionnaires covered various aspects, including basic sociodemographic information, health-seeking behaviour, morbidity details, and monthly expenses incurred on their needs. The information pertaining to their demographic details, healthcare-seeking practices, choice of health service providers, their morbidity status, and estimated monthly expenses on their needs, including medical reasons.

The qualitative data involved conducting six focus group discussions with a group of approximately five to 10 study participants. A structured discussion guide was developed, consisting of open-ended questions and prompts related to the preference for rag picking as an occupation and the reasons for substance usage among the participants. A skilled facilitator led the discussions, encouraging active participation and allowing participants to share their experiences and insights. Detailed notes were taken during the discussions to capture the participants' responses, observations, and key findings.

Data analysis approach

Data analysis involved the calculation of frequency distributions to summarise the collected quantitative data. This provided an overview of the sociodemographic characteristics, health-seeking behaviour, morbidity profile, and monthly expenses of the women rag pickers. The qualitative data involved transcription notes and analysed thematically. The identified themes and patterns related to the reasons for preference for rag picking and substance usage factors were extracted and interpreted. To ensure the reliability and validity of the findings, the data were cross-checked with multiple focus group discussions and compared to identify common themes till the saturation of responses and variations among participants.

Ethical considerations

This study has been reviewed and approved by the Institutional Review Board (IRB) of Maharashtra University of Health Sciences, Nashik (approval number: 012020). The IRB has carefully assessed the research protocol, ensuring its compliance with ethical standards and guidelines for conducting research involving human participants. The study's ethical considerations, participant welfare, confidentiality measures, and informed consent procedures have been thoroughly evaluated and found to be in alignment with the ethical principles outlined by the IRB. During the study, the participants were provided with detailed information about the study's objectives, procedures, confidentiality, and their right to withdraw from the study at any time. Informed consent was obtained from each participant prior to data collection. All collected data were kept confidential and were only accessible to the research team. Participants' identities were anonymised to ensure privacy and confidentiality.

## Results

Socio-demographic features

The study findings revealed several socio-demographic characteristics within the cohort of 150 women rag pickers. The majority of the study participants (67.3%) belonged to the age group of 15 to 30 years. Among study participants, 123 (82%) fell within the lower socio-economic class as per the modified Kuppuswamy classification, indicating a substantial prevalence of economic vulnerability among the study participants. One-third of the women had attained primary education, while the remaining were illiterate.

Approximately 43.4% of the women had tobacco usage, predominantly in the form of oral consumption. This highlights a significant prevalence of tobacco usage among women rag pickers. Over half of the family members, around 56.7%, demonstrated usage of substances such as pan, tobacco, and alcohol, indicating a high prevalence of substance usage within the households.

Health-seeking behaviour

When inquiring about the health-seeking behaviour of the study subjects, the results revealed that among the participants, 51% responded that they did not seek treatment for minor ailments or unless there was an emergency. There were 29% of the respondents who reported using home remedies or self-medications as their routine approach to address health concerns but the rest 20% stated that they approached health facilities (government or private) for any illness. Furthermore, it was noted that 80% of women surveyed indicated that they had delivered their pregnancies in healthcare facilities, indicating a utilisation of health facilities for major ailments. These results provide insights into the health-seeking behaviour patterns of the study subjects, illustrating the tendency to forgo seeking treatment for minor ailments or non-emergency situations, and the reliance on government or private hospitals for medical care.

Morbidity status among study subjects

**Figure 1:**
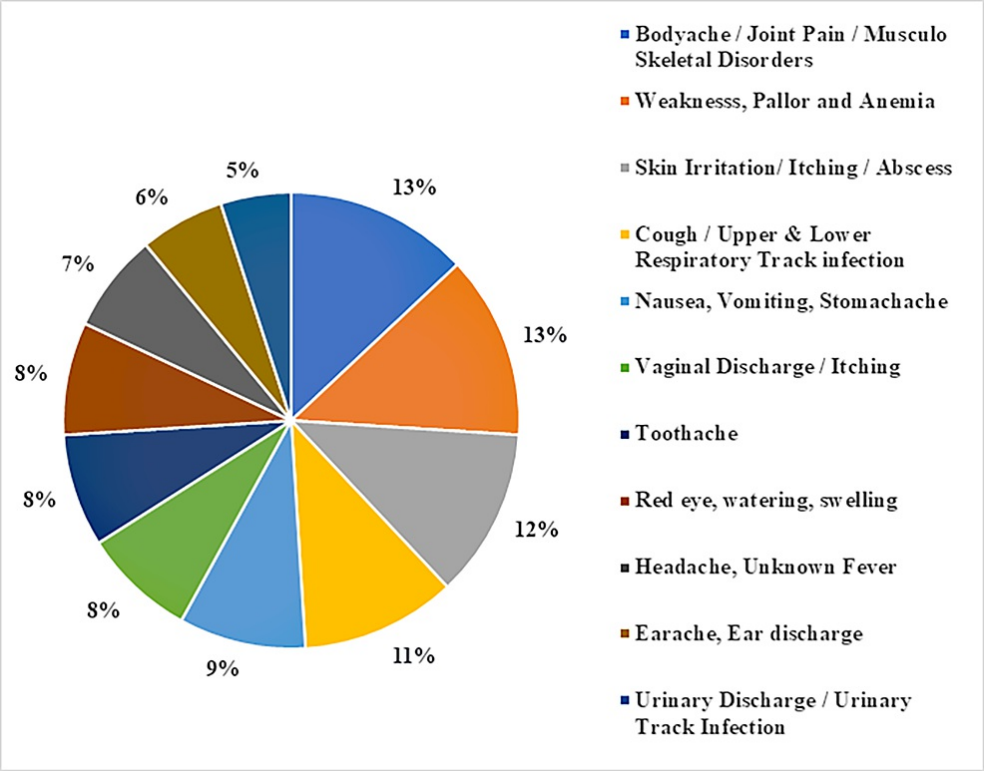
Signs and symptoms experienced in the last three months by study participants (N = 150).

The morbidity profile was assessed by inquiring about the illnesses encountered by the study participants in the last three months (Figure 1). This provided valuable insights into the prevalence and types of health conditions experienced by the population during that period, aiding in understanding the burden of morbidity and informing healthcare interventions.

Financial expense pattern observed during the study

The study found that a total of 107 (71.3%) study participants allocated most of their income towards home expenses. It was observed that 95 participants (63%) were the sole contributors of money to their respective families. The financial responsibility borne by these women was substantial, with an average of five dependent family members relying primarily on their income.

**Figure 2:**
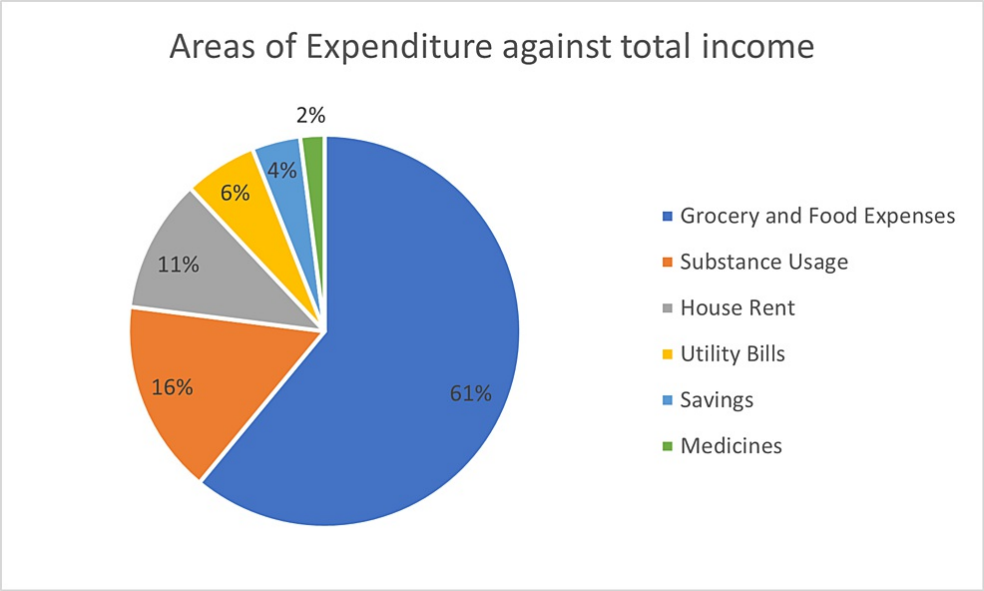
Monthly expenditure status of the study subjects (N = 150).

The study examined the monthly expenditure patterns based on the total disposable money, including income and debt. The average monthly income was approximately 9000 INR (130 USD), while the debt ranged from 500 INR to 3000 INR at the time of the interview. The study also explored the monthly expenditure patterns based on total disposable money, encompassing income and debt. Results revealed that, on average, participants allocated 61% of their income towards food expenses. These findings shed light on participants' spending habits for essential needs like food and the allocation of resources for other expenditures (Figure 2).

Findings from the qualitative research

The qualitative discussions held within focus groups involving rag pickers identified a range of findings.

Reasons for Preference of Rag Picking as an Occupation

Participants attributed the preference for rag picking as an occupation to multiple factors. One of the most critical among these was illiteracy, which significantly constrained alternative options. Concerns about risks associated with other opportunities, such as domestic work, that may create feelings of emotional abuse and false allegations. Rag picking was preferred over working for others, as it allowed for self-employment and independence. Furthermore, the findings indicated that rag-picking often emerged as a byproduct of the prevailing social exclusion system, rather than as a first-choice occupation.

"In the absence of formal education, we sought various job opportunities. Those who found something better moved on, but we have settled here. We work in people's homes, which often results in challenges, such as accusations of theft or getting mistreated."

"We do this work independently, on my own schedule. There is no external pressure. If we don't want to work on a particular day, we don't. The income in this profession may be limited, but it brings peace. If another job opportunity comes along, we would take it, but they are hard to come by. Many have tried various jobs but couldn't settle, or they were let go, so they returned." (Response received on question of having a preference for rag picking as a job)

Reasons for Substance Usage

Several factors contributing to substance usage among the participants emerged. The influence of peers played a significant role, with the company of fellow users driving personal use. Coping with stress and seeking relaxation emerged as motivations for substance usage.

"I don't know why I started, but I've been using tobacco for a while now. It became a habit since everyone around me was doing it. I haven't really thought about quitting. Sometimes, we do it for fun or to pass the time." (One of the study participants expressed her reasons for her substance usage)

## Discussion

The present study explored various aspects of the lives of women rag pickers in an Indian metropolitan city. The findings shed light on important socio-demographic features, health-seeking behaviour, morbidity status, monthly expenses, and qualitative insights into the reasons for their occupational preference and addiction [8]. Understanding these factors is crucial for developing effective interventions and policies to address the challenges faced by this vulnerable population.

Regarding the socio-demographic features, it was observed that a significant proportion of women rag pickers belonged to the lower socio-economic class, highlighting their economic disadvantage [8]. However, a positive trend was observed in healthcare utilisation, with a majority of the women reporting giving childbirth at healthcare facilities, indicating improved access to formal maternal care services. Tobacco addiction was found to be prevalent among women rag pickers, primarily in the form of oral consumption [3]. Additionally, the study revealed that these women carried a heavy responsibility as they supported an average of five dependent family members. Substance addiction was also found to be common among the family members of rag pickers, further underscoring the complex challenges faced by this population. This is similar to other studies conducted in the various demographics of metropolitan cities in India [4,9,10]. Furthermore, a low level of educational attainment was observed among women rag pickers, with one-third having attained only primary education.

The findings related to health-seeking behaviour highlighted that a considerable proportion (51%) of participants did not seek treatment for minor ailments or unless there was an emergency. Home remedies were commonly used by a significant portion (29%) of the participants as a routine approach for addressing health concerns. On the other hand, a number of participants (20%) relied on government or private hospitals when they fell ill.

These patterns indicate the need to promote regular healthcare-seeking behaviour among women rag pickers, especially for non-emergency situations, and to improve access to primary healthcare services. The existing structure of the National Health Mission covered the urban slum areas with extended services through urban primary health centres and urban Accredited Social Health Activists (ASHAs). However, there is no visible specific strategy for community mobilisation for marginalised populations to address the social determinants of health and to link them to primary care services.

The assessment of morbidity status provided valuable insights into the prevalence and types of health conditions encountered by the study participants. This information is crucial for understanding the burden of morbidity and tailoring healthcare interventions to address their specific health needs. Similar types of signs and symptoms were observed in several studies that highlight the issue to address and consider this vulnerable group to cover under comprehensive primary health care [4-6,11,12]. There were several studies indicating the need to address their health needs due to psychological vulnerability due to their work environment and financial constraints. This highlights the need for targeted mental health interventions. These groups of population may serve as vehicles for the transmission of certain pathogens that degrade waste, thereby, constituting some public health hazards. Understanding their health practices is crucial for their well-being and broader public health and creating a structure to make regular health check-up systems through the urban health centres [13,14].

The study delved into the income and spending patterns of women engaged in rag picking. On average, they earned approximately 9000 INR (equivalent to 130 USD) each month, with a significant portion, around 61%, allocated for food expenses. Interestingly, health care was not a top priority for these women, as reflected in their limited medical expenditures. These findings underscore the financial challenges these women encounter and emphasise the importance of ensuring their access to affordable healthcare services related to their occupational health hazards [8,10,15].

Qualitative interviews revealed several factors influencing the preference for rag picking as an occupation and the reasons for substance usage among participants. Concerns about risks associated with other opportunities, such as domestic help, influenced their preference for self-employment. Numerous studies have consistently echoed these findings, emphasizing that the preference for rag-picking often arises due to limited alternative job opportunities within the context of social exclusion. The role of peer influence in driving personal substance use, along with the utilisation of substances as a coping mechanism, has been a recurrent theme among the participants [8,16].

The work done by waste pickers often goes unnoticed, but they play an important role in society by saving money on waste collection and disposal. Many studies have tried to figure out how much money the informal waste sector contributes to the economy - it is a lot, billions of dollars each year. A closer look by Medina shows that waste pickers actually help society in positive ways, and with some help, they could do even more [17]. Plus, waste picking also helps save money by making waste management and disposal cheaper. Further detailed studies on their hygiene practices, non-use of protective equipment, inhuman living conditions, and level of health awareness will provide a critical understanding while making social and health interventions to recognise their contribution to our health environment.

Limitations

Some potential limitations of this study were the use of self-reported data, which may be subject to recall bias, and the cross-sectional design, which limits the ability to establish causal relationships between variables.

## Conclusions

In conclusion, this mixed-methods study provided comprehensive insights into the lives of women rag pickers, encompassing socio-demographic features, health-seeking behaviour, morbidity status, monthly expenses, and qualitative findings. These findings contributed to the existing knowledge and can support in development of targeted interventions aimed at improving the well-being and socio-economic conditions of women rag pickers. The universal coverage of health programs, provision, and inclusion of this vulnerable group for health awareness to ensure right health-seeking behaviour and accessible community-based social interventions should be focused to ensure the well-being and betterment of women rag pickers in India.
